# Successful Treatment of Ulcerative Senile Gluteal Dermatosis by the Concept of Skin Barrier Recovery: A Case Report and Literature Review

**DOI:** 10.7759/cureus.114863

**Published:** 2026-08-20

**Authors:** Yijiao Wang, Qiannan Xu

**Affiliations:** 1 Wound Care, Lujiazui Community Health Service Center, Shanghai, CHN; 2 Dermatology, Shanghai East Hospital, Shanghai, CHN

**Keywords:** individualized nursing, medical economics, pressure offloading, senile gluteal dermatosis, skin barrier repair, ulcerative sgd

## Abstract

Senile gluteal dermatosis (SGD) is an underrecognized friction- and pressure-related dermatosis predominantly affecting elderly individuals with prolonged sitting and low BMI. It typically presents as hyperkeratotic, lichenified plaques with horizontal ridges within the “sitter’s triangle” (the area bounded by the coccygeal apex and the two ischial tuberosities), with or without ulceration. We report the case of a 68-year-old Chinese man with a three-year history of pruritic hyperkeratotic plaques on the left buttock that progressed to superficial ulceration after repeated scratching. Clinical features were characteristic of ulcerative SGD. After gentle debridement of scales and application of a foam dressing aimed at skin barrier restoration, along with pressure off-loading, complete epithelialization occurred within five days, with subsequent fading of residual pigmentation. This case highlights that targeting skin barrier repair could be a potentially effective core strategy for treating SGD.

## Introduction

Senile gluteal dermatosis (SGD), first described in Japan in 1979 [1], is characterized by vaguely circumscribed brownish or erythematous hyperkeratotic plaques with fine, parallel, horizontal ridges, classically distributed in a “three-corners-of-a-triangle” pattern over the gluteal cleft and bilateral buttocks [2,3]. The condition is strongly associated with advanced age, prolonged sitting, and friction against hard surfaces [4-6].

Histologically, findings are nonspecific and include hyperkeratosis, psoriasiform epidermal hyperplasia, and dermal vascular proliferation; amyloid is typically absent, distinguishing SGD from anogenital cutaneous amyloidosis and pressure injury [7-9]. Dermoscopy showed that SGD displayed a mixed vascular pattern, with dotted, linear, and linear-curved vessels surrounded by a white halo and distributed uniformly, with follicular plugs often surrounded by the above-mentioned vessels [10,11]. Recent literature has proposed the unifying term “reactive epidermal hyperplasia and angiogenesis of the rear (REAR)” to emphasize the dual epidermal and vascular response to chronic mechanical stress within the sitter’s triangle [12]. Although usually asymptomatic or mildly symptomatic, SGD may progress to erosion or shallow ulceration, which might be complicated by scratching or prolonged pressure [13]. Differential diagnoses include lichen simplex chronicus, inverse psoriasis, and early pressure injury [14,15].

Treatment centers on pressure off-loading and skin barrier support; topical corticosteroids and keratolytics often yield limited benefit [4,5,14]. We present a case of ulcerative SGD successfully managed by targeted skin barrier repair with a foam dressing, achieving rapid healing without pharmacologic intervention.

## Case presentation

**Figure 1:**
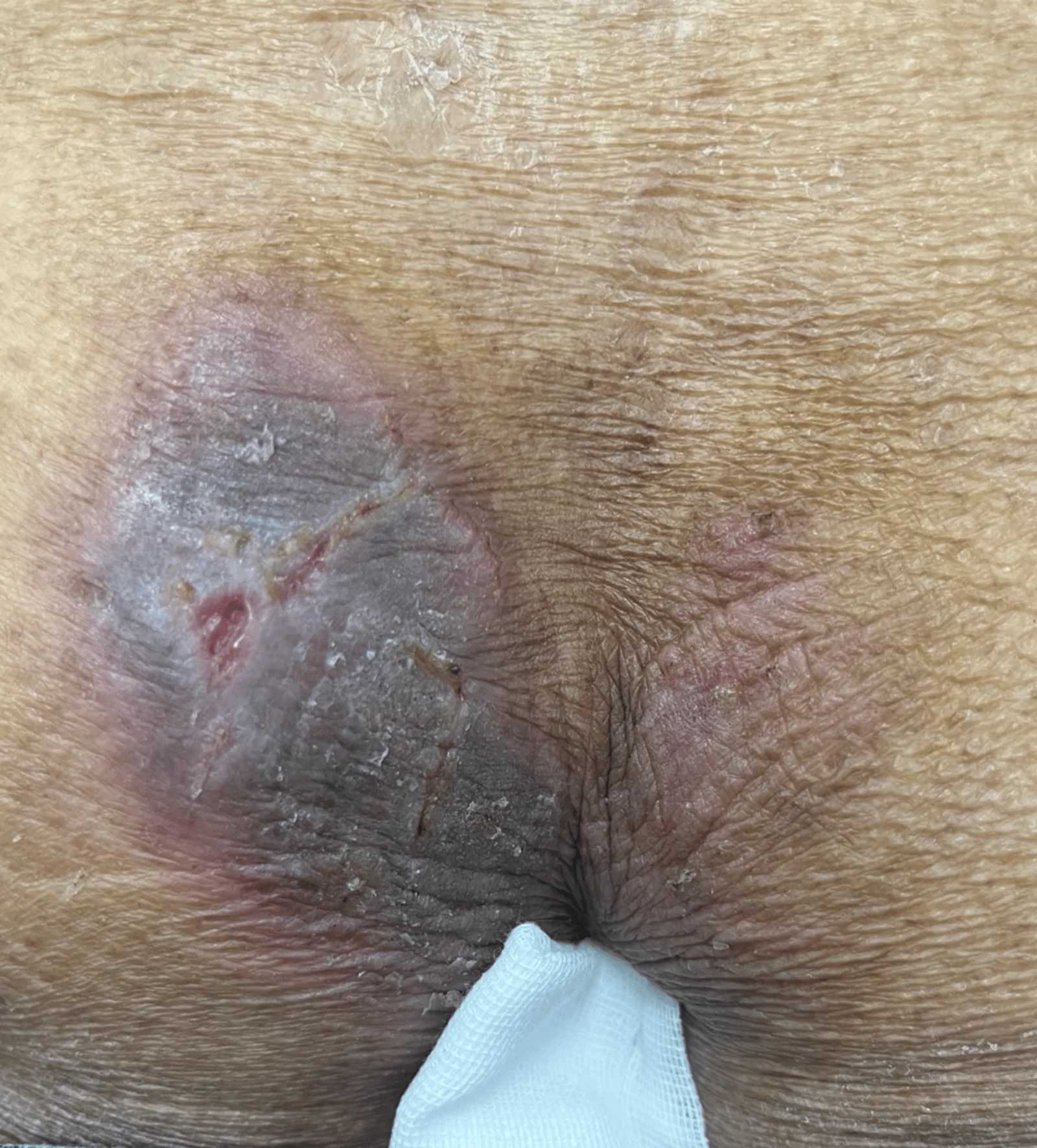
Skin lesion before treatment.

A 68-year-old Chinese man presented to the wound care clinic with a three-year history of intermittent pruritus over the left buttock. He reported frequent prolonged sitting (>6 hours daily), including midday napping while seated. Physical examination revealed a 5 × 5 cm area of hyperkeratotic, rough, brownish, scaly plaque on the left buttock with prominent horizontal ridges, consistent with the sitter’s triangle distribution. After gentle cleansing and scale removal, a shallow ulcerated base was exposed (Figure 1). He had a history of hypertension and coronary heart disease but no history of smoking or alcohol use.

Dermoscopy showed mixed vessels, follicular plugs, and white halos, and the diagnosis of SGD was confirmed. Although the onset of the ulcer was unclear, the white halo provided strong evidence that this 0.2-cm-deep ulcer might have been present for months without treatment. His BMI was 20 kg/m² (within the normal range). There was no history of diabetes, incontinence, or other comorbidities. Systemic examination was unremarkable. Biopsy was deferred given the classic presentation and the patient’s preference to avoid additional trauma. Treatment consisted of gentle mechanical debridement of residual scale, disinfection, and application of a nonadherent foam dressing to provide moisture balance, cushioning, and barrier protection. The patient was instructed to reduce continuous sitting time, use a pressure-relieving cushion, and apply emollients after dressing changes.

**Figure 2:**
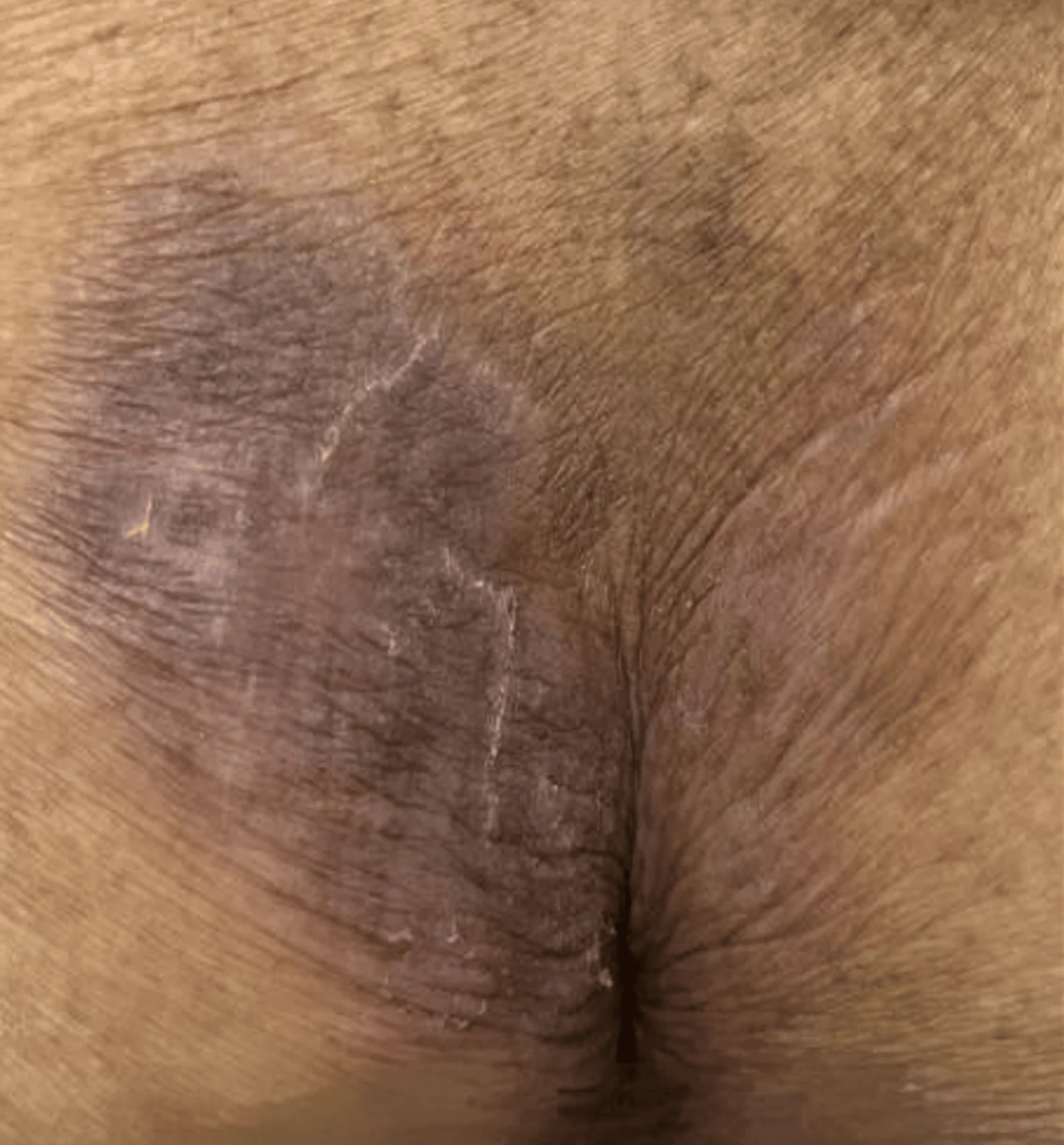
Epithelialization after five days of skin barrier repair treatment.

At the five-day follow-up, the ulcer had completely epithelialized (Figure 2). Residual post-inflammatory pigmentation was noted but continued to lighten over the subsequent weeks with ongoing lifestyle modification and moisturization. Short-term clinical follow-up at four weeks and three months confirmed no recurrence. 

## Discussion

SGD is increasingly recognized in East Asian populations, with a reported prevalence of approximately 13% among individuals older than 60 years [5]. The pathogenesis is attributed to repetitive friction, shear, and microischemia over bony prominences, triggering epidermal hyperplasia and reactive dermal angiogenesis [14]. Mechanical stress due to prolonged sitting, which exceeds the skin’s reparative capacity, especially in thin elderly men, was considered to be a key factor in SGD formation and ulceration [6]. However, most cases showed that patients with SGD had a normal BMI (Table 1).

**Table 1:** Summary of clinical features, treatment methods, and outcomes of SGD cases in the literature. SGD, senile gluteal dermatosis

Source	Number of cases	Age (mean/range)	Sex ratio (M:F)	BMI (mean)	Lesion morphology and location	Main symptoms	Main treatment	Outcome
Liu et al. (2014) [4]	137	79.4 years	130:07:00	21.7	Typical sitter’s triangle + horizontal ridges, some ulcers	Pruritus/pain (36%/10%)	Pressure relief + corticosteroids (moderate effect)	Improved
Moon et al. (2016) [5]	37	70.4 years (60-92)	17:20	22.76	Typical triangle area, possible ulcers	Mild or asymptomatic	Lifestyle intervention	Approximately two months
Tang et al. (2024) [6]	36	84.2 years	2:01	21.7	Brownish scaly patches + erythema + ulcers	Pruritus/discomfort	Pressure-relief cushion + salicylic acid + moisturizer	Curable
Garbayo-Salmons and Exposito-Serrano (2024) [9]	4	70-85 years	2:02	29.3-37	Gluteal ulcers/hyperkeratosis (ulcerative)	Pruritus/pain + itch	Pressure relief	Partial improvement (with lumbar abnormality)
Cebolla-Verdugo et al. (2024) [11]	1	83 years, M	-	Not reported	Intergluteal cleft + bilateral buttocks: reddish-brown to gray hyperkeratotic scaly plaques	Mild pruritus	Topical retinoid + posture hygiene education	Marked improvement in three to six months
Liang et al. (2022) [12]	4	68-73 years	Predominantly male	Not reported	Sitter’s triangle erythematous scaly plaques + horizontal hyperkeratotic ridges	Discomfort when sitting	Lifestyle intervention (mainly pressure relief)	Improved
Gwak et al. (2017) [13]	7	Not detailed	Not detailed	Not detailed	Ulcerative SGD	Pruritus/pain	Mainly pressure relief	Improved
Wu and Chong (2022) [15]	1	69 years, M	-	Not reported	Bilateral buttocks + medial ischial tuberosities + gluteal cleft: erythematous lichenoid + horizontal ridges + erosion	Hot pain when sitting	Pressure-relief cushion + topical calcipotriol	Significant improvement in three months

Some studies have suggested that SGD might be associated with culture, as Asians tend to use hardwood chairs [6]. However, data on sex provide equivocal support. Liu et al. showed only seven female cases among 137 SGD cases in a study conducted in China, whereas Korea, also an Asian country, had 20 female cases among all 37 SGD cases. China and Korea share a similar Asian culture, which means that if sitting behavior plays a major role in the formation of SGD, the cases should show a similar sex distribution among different populations [4]. Thus, although in our case the patient was asked to avoid prolonged sitting on a chair, this might not have been the most important factor contributing to the rapid five-day recovery.

The frictional and shear forces resulting from prolonged sitting are widespread among the elderly and even among middle-aged and young populations. However, not all individuals who sit for long periods will develop SGD. If the skin barrier function remains intact and the structure is sound, simple physical and mechanical forces are insufficient to trigger this disease. The skin can completely accommodate stress through normal compensatory mechanisms. Only when the skin barrier is damaged and the “brick wall structure” of the stratum corneum becomes unstable can mechanical stress continuously stimulate the epidermis, prompting keratinocytes to release cytokines such as vascular endothelial growth factor, thereby activating reactive epidermal proliferation and dermal angiogenesis (i.e., the REAR phenomenon) [14].

Majid et al. acknowledged that emollients are “essential” for SGD management and described “dry skin” as a clinical feature, as well as “breakdown of the skin barrier” leading to secondary infection [14]. These observations align with the view that skin barrier dysfunction is not merely a secondary consequence but a central pathogenic defect. While mechanical triggers such as prolonged sitting may initiate the process, the persistence and clinical expression of SGD depend critically on the integrity of the stratum corneum.

Our case, which achieved rapid healing through barrier-focused therapy without prolonged pressure off-loading, supports this interpretation. Therefore, we believe that mechanical stress and lifestyle habits are merely “catalysts” or “environmental variables” for the occurrence of SGD, while damage to the skin barrier is the core reason for the occurrence of SGD. Establishing this mechanism directly supports the principle that clinical intervention should take “skin barrier repair” as the core principle [6].

Topical retinoids or vitamin D analogues have shown benefit in selected reports, yet most required months of treatment to achieve recovery. In our case, simple moisture-balanced dressings achieved rapid epithelialization. Foam dressings simultaneously protect the fragile barrier, maintain a moist wound environment [16], and provide modest cushioning, making them particularly suited to the shallow ulcers of SGD. Our case illustrates that ulcerative SGD responds promptly once the dual insults of friction and barrier disruption are addressed. Since pressure off-loading was used in all SGD treatments reported in the literature, the rapid five-day recovery could be attributed to skin barrier recovery. Moreover, with rapid recovery, patients do not have to change their habits for long, which may be difficult for elderly individuals.

## Conclusions

This case illustrates that a nurse-led, individualized model combining skin barrier repair, pressure off-loading, and targeted education can achieve rapid healing and prevent misdiagnosis. More cases may provide corroborative evidence regarding the optimal treatment approach, especially treatment without pressure off-loading. While further controlled studies are warranted, this practical paradigm offers value for community-based chronic skin disease management.
